# The potential of Beta variant containing COVID booster vaccines for chasing Omicron in 2022

**DOI:** 10.1038/s41467-022-33549-6

**Published:** 2022-10-02

**Authors:** Saranya Sridhar, Roman M. Chicz, William Warren, Jim Tartaglia, Stephen Savarino, Sanjay Gurunathan, Jean-Francois Toussaint

**Affiliations:** 1 Sanofi, Reading, UK; 2 Sanofi, Waltham, US; 3 Sanofi, Swiftwater, PA USA; 4 Sanofi, Orlando, US; 5 Sanofi, Lyon, France

**Keywords:** Vaccines, SARS-CoV-2

## Abstract

Currently approved COVID vaccines are designed using the spike antigen derived from the ancestral strain, but health authorities are recommending changes to the vaccine strain to combat emerging variants. The goal is to ensure that next generation vaccines can tackle multiple variants of concern including the most prevalent variant for the coming season. We here discuss recent preclinical and clinical data on COVID vaccine antigens that are potential candidates for an updated vaccine.

SARS-CoV-2 variants of concern (VOC) continue to emerge, largely because some errors that are introduced during virus replication can be advantageous in regards to viral fitness and/or immune escape. One approach to tackle emerging VOCs is to focus booster vaccines on the predominantly circulating variant, which is currently Omicron^1^. This would mimic what has been done successfully with vaccines based on the ancestral SARS-CoV-2 strain. However, an alternative approach could be to select an antigen that induces broad cross-reactivity to several VOCs, including the currently dominant variant. Here, we discuss preclinical and clinical evidence supporting the relevance of a booster vaccine based on SARS-CoV-2 Beta (B.1.351) spike protein and speculate on potential reasons for broad cross-reactivity of a Beta-based booster vaccine. We argue that several options for strain selection for the next booster should be considered, and that each strain option must be justified by scientific evidence.

## The virus has changed—so has the population’s immune status

In 2020 and in the first half of 2021, COVID-19 vaccines aimed at building a protective immune response in populations that had never been exposed to the SARS-CoV-2 virus and that presented virtually no meaningful pre-existing immunity. While pre-pandemic samples from individuals with evidence of exposure to seasonal human coronaviruses have exhibited SARS-CoV-2 cross-reactive CD4 + T cells and non-neutralizing antibodies primarily directed against SARS-CoV-2 nucleocapsid and spike protein S2 domain, there is conflicting evidence as to whether this mitigated COVID-19 disease severity^2–5^. The circulating virus strains closely matched the vaccine sequence and the immune response induced by the vaccines were protective with high efficacy and effectiveness against symptomatic and severe infection^6,7^. The emergence of new variants has led to the initially observed high efficacy and effectiveness against symptomatic COVID degrading and becoming more variable. Over time, a more complex and heterogeneous immune status has emerged in the population, shaped by infection from variant virus strains and  the exposure to vaccines. In this new complex virological and serological reality^8^, the booster vaccines that should protect against a potential surge of SARS-CoV-2 in the autumn and winter of 2022 have the challenging task to reshape the existing immune response, boost its intensity and increase its breadth to offer protection against newly emerging VOCs, including Omicron and its subvariants. However, what brought robust vaccine efficacy against symptomatic and severe COVID-19 in the early stages of the pandemic may not translate into the needed booster response going forward.

Recent data suggest that some vaccine candidates that efficiently primed the immune response did not offer the best booster response. For example, an inactivated vaccine induced a robust immune response as a primary vaccination schedule in naïve individuals but failed to efficiently boost the immune response in people with pre-existing immune responses^9,10^. Other vaccines, like mRNA-1273 or BNT162b2 have had good performance both in primary regimens^11,12^ and as a booster^10,13^

## Beta-based booster vaccines in animal models and humans

The spike protein sequence and design are key determinants to broaden the immune response elicited in people with pre-existing immunity. In the face of the emerging epidemic of the Beta variant (B.1.351) in South Africa in May 2021, we and others tested vaccine candidates based on the Beta variant spike in preclinical models and clinical trials. In non-human primates previously primed with an ancestral strain spike antigen, Beta variant-containing mRNA and adjuvanted recombinant protein vaccines have shown broad cross-reactivity across SARS-CoV-2 VOCs and SARS-CoV-1^14,15^. A Phase 2/3 study of a bivalent mRNA vaccine encoding spike antigen of Beta variant and D614 ancestral strain also showed higher levels of cross-reactive immune responses across VOCs 6 months post-boost compared to that of the corresponding vaccine based on the ancestral strain^16^. In a recent clinical trial conducted in SARS-CoV-2 naïve individuals in France comparing monovalent recombinant protein prototype and Beta-variant booster vaccines with AS03 adjuvant to the BTN162b2 mRNA prototype booster vaccine, the Beta-variant booster vaccine showed broader cross-reactive neutralizing antibodies across VOCs including Omicron, implicating the modified antigen rather than the adjuvant in this effect^17^. In this study, the Beta booster vaccine candidate boosted Omicron BA.1-specific neutralizing antibody titers 14.6-fold compared to baseline and 1.82-fold higher than after the prototype mRNA vaccine BNT162b2.

A critical question is the relative performance of a Beta-booster vaccine compared to Omicron-containing booster vaccines. To the best of our knowledge and at the time of writing, a direct comparison of both vaccine candidates has not been done or published and comparison of results across trials should be considered with caution due to differences in populations, timing and neutralization assays. Nevertheless, to put the results obtained with Beta-based boosters into perspective, it is of interest to provide recent results obtained with Omicron containing booster vaccine candidates. An mRNA vaccine based on the Omicron spike sequence compared to the same vaccine based on the prototype strain failed to provide a better boost of the Omicron-specific titers in non-human primates^18^. Recent clinical data shows that the Omicron BA.1 containing bivalent mRNA-1273.214 booster vaccine induced an 8-fold increase of Omicron-specific antibody titers compared to baseline and 1.75-fold higher Omicron-titers compared to the mRNA-1273 vaccine based on the ancestral strain alone^19^. This challenges the conventional wisdom that increasing valencies in a vaccine formulation improves breadth. Additionally, a modified Omicron-specific BNT162b2 monovalent mRNA vaccine (BNT162b2 OMI) at a 30 or 60 μg dose, elicited a 13–20-fold increase in BA.1 neutralizing antibody titers compared to baseline in SARS-CoV-2 naïves, which was 2–3 fold higher than BNT162b2^20^.

Altogether, and while important data are still awaited with Beta and Omicron-containing vaccine candidates, the currently available results summarized above lead us to believe that booster vaccines based on the spike antigen from the Beta variant virus are relevant candidates for upcoming booster campaigns.

## Considerations for induction of cross-neutralizing antibodies by Beta-variant booster vaccines

The history of influenza vaccine strain selection suggests that selecting a variant vaccine strain that matches the most predominant circulating strain may not be an optimal solution. The one principle that underpins the process is that the selected strain, whether prevalent or not, must provide protection against the predominant circulating strain causing symptomatic and severe disease. The most prevalent strains can suffer from survivorship bias limiting their use as immunogen because of two general features: (1) highly cross-reactive strains may extinguish in the population and not necessarily be prevalent and (2) most immunogenic viral strains build up herd immunity that limit their survival (assuming the most commonly held framework of population dynamics of antigenically variable pathogens such as influenza). There is some evidence that supports the notion that the highly prevalent Omicron VOC may suffer from this bias. Omicron VOC has been circulating for an extended period of time (spanning more than 7 months); Omicron infections are associated with substantially lower cross-reactive antibody titers than other VOC infections^21^; and Omicron spike has been reported to show reduced antigenicity^22^. On this basis, optimal booster vaccines may preferably rely on other strains, which exhibit broad cross-reactivity patterns in humans against current circulating strains and are highly immunogenic and are relevant against past and future strains likely to cause the most severe disease.

As populations increasingly experience multiple SARS-CoV-2 exposures, it will be important to understand immunodominance patterns and how epitopes targeted by vaccination with new variants may differ from those inferred from first exposure and from circulating strains. Key insights linking sequence mutations to antigenicity reveal that major antigenic differences are caused by substitutions at positions 417, 452, 484, and possibly 501 in the receptor binding domain (RBD) of the spike protein, with the Omicron strains showing the highest immune escape. Variants like Omicron and Beta with substitutions at position 484 (E to K or Q) have shown poor neutralization by convalescent sera collected from individuals infected with D614G (prototype strains), B.1.1.7 (alpha variant), B.1.617.2 (delta variant), but not with the B.1.351 (Beta) variant^23^ consistent with our discussion above.

Substitutions in the Beta variant spike at positions K417N, E484K, N501Y provide new antibody epitopes which are well-positioned to provide cross-neutralizing immunogenicity against a wide array of variants including contemporary circulating strains. Compared to Beta, Omicron has a larger antigenic distance with a lower number of shared epitopes with other VOCs^23^, forcing the immune response to focus on a narrower epitope range and causing the observed lower cross-reactive antibody titers against other VOCs^19^.

The other mutations in the Beta variant spike antigen (NTD, S1 and S2 domains) are also important as they may contribute to stabilizing the S antigen structure primarily in the open or “up” conformation exposing the RBD antibody epitopes to elicit an expanded breadth of humoral response upon boosting with this strain. Class 1 epitopes tend to be less exposed when the RBD is locked in the “down” position. Structural characterization of the B.1.351 (Beta) S antigen shows a preferred conformation (>80%) in the open or “up” position^24^. Cryoelectron microscopy studies of our Beta immunogen confirm that more than 80% of the recombinant protein remains in the open or “up” position, a substantial increase from what we observed with our ancestral strain-based vaccine candidate. Whether the reduced antigenicity observed with Omicron spike reflects more dynamic conformational structure of the spike protein and/or the 32 mutations in the RBD region remains to be investigated.

## Conclusion

SARS-CoV-2 vaccines based on the ancestral strain offer limited and rapidly waning protection against Omicron COVID-19 disease, prompting health authorities to solicit more effective, modified booster vaccines to use in the autumn and winter of 2022. Some regulatory authorities have been prescriptive about the need for Omicron BA.4/BA.5-prototype bivalent vaccines, whereas other regulatory authorities have suggested a broader approach.^25^ While the available clinical data on BA.1-containing mRNA vaccines indicate superior neutralizing responses to BA.1 compared to the corresponding prototype vaccines, this sublineage has receded, and clinical data with BA.4/BA.5 -prototype vaccines are being generated. We and others have accrued evidence supporting the value of SARS-CoV-2 vaccines based on the spike of Beta variant to boost cross-neutralizing antibodies against known variants of concerns, including Omicron.

As the virus continues to evolve and in the absence of full understanding of the determinants for the selection of the optimal sequence, we advocate for a booster strain selection strategy that is based on the greatest cross-neutralization data in humans. Coming back to our original postulate that each vaccine candidate must provide data-driven decisions to justify their booster strain selection strategy, we believe that the data indicate that the Beta VOC should be a contender for booster SARS-CoV-2 vaccine.
